# Longitudinal tumor fraction trajectories predict risk of progression in metastatic HR^+^ breast cancer patients undergoing CDK4/6 treatment

**DOI:** 10.1002/1878-0261.12870

**Published:** 2020-12-18

**Authors:** Nadia Dandachi, Florian Posch, Ricarda Graf, Christoph Suppan, Eva Valentina Klocker, Hannah Deborah Müller, Jörg Lindenmann, Angelika Terbuch, Ellen Heitzer, Marija Balic

**Affiliations:** ^1^ Division of Oncology Department of Internal Medicine Medical University of Graz Austria; ^2^ Research Unit Epigenetic and Genetic Cancer Biomarkers Medical University of Graz Austria; ^3^ Institute of Human Genetics Diagnostic and Research Center for Molecular BioMedicine Christian Doppler Laboratory for Liquid Biopsies for early Detection of Cancer Medical University of Graz Austria; ^4^ Divison of Thoracic and Hyperbaric Surgery Department of Surgery Medical University of Graz Austria; ^5^ Research Unit Circulating Tumor Cells and Cancer Stem Cells Medical University of Graz Austria

**Keywords:** CDK4/6 therapy, circulating tumor DNA, joint model, longitudinal biomarker analysis, metastatic breast cancer

## Abstract

Despite improved clinical outcomes, intrinsic or acquired resistance to CDK4/6 inhibitor treatment has limited the success of this treatment in HR^+^HER2^−^ metastatic breast cancer patients. Biomarkers are urgently needed, and longitudinal biomarker measurements may harbor more dynamic predictive and prognostic information compared to single time point measurements. The aim of this study was to explore the longitudinal evolution of circulating tumor fractions within cell‐free DNA assessed by an untargeted sequencing approach during CDK4/6 therapy and to quantify the potential association between longitudinal *z*‐score measurements and clinical outcome by using joint models. Forty‐nine HR^+^HER2^−^ metastatic breast cancer patients were enrolled, and *z*‐score levels were measured at baseline and during 132 follow‐up visits (median number of measurements per patient = 3, 25^th^–75^th^ percentile: 3–5, range: 1–8). We observed higher baseline *z*‐score levels (estimated difference 0.57, 95% CI: 0.147–0.983, *P*‐value = 0.008) and a constant increase of *z*‐score levels over follow‐up time (overall *P*‐value for difference in log *z*‐score over time = 0.024) in patients who developed progressive disease. Importantly, the joint model revealed that elevated *z*‐score trajectories were significantly associated with higher progression risk (HR of log *z*‐score at any time of follow‐up = 3.3, 95% CI, 1.44–7.55, *P* = 0.005). In contrast, single *z*‐score measurement at CDK4/6 inhibitor treatment start did not predict risk of progression. In this prospective study, we demonstrate proof‐of‐concept that longitudinal *z*‐score trajectories rather than single time point measurements may harbor important dynamic information on the development of disease progression in HR^+^HER2^−^ breast cancer patients undergoing CDK4/6 inhibitor treatment.

## Graphical abstract

With longitudinal untargeted assessment of circulating tumor DNA in metastatic HR^+^ breast cancer patients during CDK4/6 treatment and joint model analyses, we demonstrated that tumor fraction trajectories rather than single time point measurements provide important dynamic information on developing disease progression. The joint model can also use the trajectories for providing dynamic predictions for the individual patient.

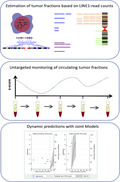

**Abbreviations**
CDK4/6: cyclin‐dependent kinase 4/6
CI: confidence interval
ctDNA: circulating tumor DNA
HER2: human epidermal growth factor 2
HR: hazard ratio
HR: hormone receptor
mFAST‐SeqS: modified Fast Aneuploidy Screening Test‐Sequencing System
OS: overall survival
PFS: progression‐free survival

## 1 Introduction

With the introduction of cyclin‐dependent kinase 4/6 (CDK4/6) inhibitors, the treatment of metastatic hormone‐receptor (HR)‐positive, human epidermal growth factor 2 (HER2)‐negative (HR^+^HER2^−^) breast cancer has made substantial progress and the combination of endocrine therapy with CDK4/6 inhibitors now prevails in the management of advanced HR‐positive breast cancer [1]. However, success has been limited despite improved clinical outcomes, as ˜ 20% of patients will not respond to CDK4/6 inhibitors initially, and the majority of patients ultimately develop treatment resistance over time with either *de novo* or acquired resistance [2, 3]. Therefore, a key challenge remains in identifying prognostic and predictive biomarkers in order to optimize treatment in clinical practice and ultimately improve outcomes in this patient group.

Liquid biopsy, in particular circulating tumor DNA (ctDNA), is being used increasingly for disease monitoring and therapy selection in advanced cancer stages [4, 5]. Beyond mutational analysis, ctDNA provides real‐time monitoring of tumor burden, since it reflects bulk DNA from different tumor locations [6, 7]. We have previously demonstrated the prognostic utility of ctDNA levels detected with modified Fast Aneuploidy Screening Test‐Sequencing System (mFAST‐SeqS) as a very fast and cost‐effective method to assess the ctDNA fraction in plasma samples without prior knowledge of the genetic landscape of the tumor [8].

Cancer is a dynamic process accompanied by cancer evolution and acquired resistance to treatment [9], but disease progression outcomes may also be influenced by a variety of non‐tumoral factors related to treatment, intercurrent infections, comorbidity, and performance status. Similarly, biomarker levels such as tumor fractions and ctDNA levels may vary over time in relation to these factors. Thus, it is conceivable that a single biomarker measurement may only represent one snapshot in time of a complex and dynamic disease, and that a string of biomarker measurements over time (i.e., a longitudinal trajectory) may harbor more prognostic and predictive information. With ctDNA as a surrogate marker of disease burden, we expect that tumor fractions measured in blood by mFAST‐SeqS change over time during cancer treatment and we hypothesize that assessment of ctDNA trajectories may have dynamic prognostic information on clinical outcome beyond a single measurement in time.

In this context, so‐called joint models that combine longitudinal and time‐to‐event data have been developed and used for examining this kind of research question [10, 11, 12]. As such, the aim of this study was to explore the longitudinal evolution of *z*‐scores using mFAST‐SeqS during CDK4/6 therapy using joint models and to quantify the potential association between longitudinal *z*‐score measurements and clinical outcome. We also assessed the prognostic association between baseline *z*‐scores at CDK4/6 treatment start and clinical outcome.

## 2 Materials and methods

### 2.1 Study design and patients

In this prospective, pilot proof‐of‐concept, observational study, we included all 49 patients with advanced or metastatic HR^+^HER2^−^ breast cancer with measurable disease that were scheduled for treatment with CDK4/6 inhibitors (Palbociclib: *n* = 36, Ribociclib: *n* = 8, Abemaciclib: *n* = 5) and antihormonal therapy at the Division of Oncology, Medical University of Graz between February 2017 and March 2020. The study was approved by the ethics committee of the Medical University of Graz (ethical approval number 21–227 ex 09/10), and written informed consent was obtained from all patients. This research was carried out according to the principles of the Declaration of Helsinki and complied with reporting recommendations for tumor marker prognostic studies (REMARK) criteria [13] (Table S1). Baseline data on patient demographics, tumor characteristics, treatment data, and clinical outcome were retrieved from the in‐house electronic healthcare database and from paper charts. Information on survival status was collected from the central registry of the Austrian Social Security Providers Association. Blood samples were obtained before start of CDK4/6 treatment (baseline sample) and at follow‐up visits, consistent with routine standard clinical care adapted from clinical studies performed with CDK 4/6 inhibitors, every 2–3 months or as indicated by the treating physician until disease progression or death. Patients underwent periodic imaging and treatment response assessment according to standard response evaluation criteria in solid tumors 1.1. Due to the exploratory nature of the current study and the difficulty of power estimation for joint modeling, no formal sample size calculation was performed.

Our primary interest was to investigate the association between the longitudinal trajectories of *z*‐score levels and the clinical outcome as represented by progression‐free survival (PFS). The PFS was defined as the time from first blood draw (before treatment start) until disease progression, censoring alive, or death, whichever occurred first. Overall survival (OS) was a secondary endpoint and was defined as the time from first blood draw until censoring alive or death‐from‐any‐cause.

### 2.2 Plasma isolation and mFAST‐SeqS

Isolation of plasma DNA was performed as previously described [14]. Briefly, whole blood (9–18 mL) was collected in PAXgene Blood ccfDNA Tubes (Qiagen, Hilden, Germany). Plasma was isolated from blood samples by centrifugation at 200 ***g*** for 10 min followed by 1600 ***g*** for 10 min with brake and acceleration powers set to slow. The supernatant was then removed and centrifuged again at 1600 ***g*** for an additional 10 min. Plasma was then stored in 2 mL tubes at −80 °C. Plasma DNA was isolated from 2 mL plasma using the QIAamp Circulating Nucleic Acid Kit (Qiagen) according to the manufacturer's recommendations or using the QIASymphony PAXgene Blood ccfDNA Kit (Qiagen). Qubit dsDNA HS Assay Kit (Life Technologies, Vienna, Austria) was used for quantification of plasma DNA.

LINE1‐amplicon libraries for mFAST‐SeqS were prepared as previously described [15]. Briefly, 0.5–2 ng of plasma DNA was amplified with Phusion Hot Start II Polymerase in eight PCR cycles using target‐specific L1 primers. The 10 µL of the purified (AMPure Beads, Beckman Coulter, Brea, CA, USA) PCR products was used for the second PCR in which Illumina‐specific adaptors and indices were added. L1 amplicon libraries were pooled equimolarly and sequenced on an Illumina MiSeq or NextSeq, generating 150 bp single reads or 76 bp paired‐end reads aiming for at least 80 000 reads per sample. Sequence reads were aligned to the hg19 genome, and reads with a mapping quality > 15 were counted per chromosome arm using an in‐house script. Normalized read counts, that is, read counts per chromosome arm scaled by total number of reads, were compared to a control population (*n* = 35) using *z*‐score statistics by subtracting the mean and dividing by the standard deviation. This assessment of over‐ and under‐representation of LINE1‐sequences provides a proxy for the presence of gains or losses of chromosomal material on a chromosome‐arm level. The short arms of acrocentric chromosomes were omitted from the analysis. Finally, all chromosome‐arm‐specific *z*‐scores were squared and summed up, resulting in a genome‐wide *z*‐score, which acts as a surrogate for tumor fraction [16].

### 2.3 Statistical analyses

All statistical analyses were performed with Stata 16.1 (Stata Corp., Houston, TX, USA). Continuous variables were reported as medians [25th–75th percentile] and count data as absolute frequencies (%). In all analyses, we used log‐transformed *z*‐scores to account for its skewed distribution. In addition, *z*‐score levels were dichotomized into a binary variable (< 3 and ≥ 3) as previously published by our group [8]. Median follow‐up time was estimated with the reverse Kaplan–Meier estimator [17]. The distribution of baseline variables between patients with high or low *z*‐score levels at baseline (binary *z*‐score variable) was assessed with rank‐sum tests (continuous variables) and chi‐squared, or with Fisher's exact tests (categorical variables). The association between *z*‐score levels at baseline and clinical outcomes was evaluated with Kaplan–Meier estimators, log‐rank tests, and Cox proportional hazards regression. The proportional hazards assumption was tested using Schoenfeld tests. The longitudinal change in *z*‐score over time was analyzed with a linear mixed model, using a quadratic growth model for follow‐up time and a random intercept at the patient level and a random slope for linear follow‐up time. Joint models for longitudinal and time‐to‐event data were used for quantifying the relationship between *z*‐score trajectories and clinical outcomes [18]. The joint model was specified as follows: (a) A quadratic mixed growth model with random intercept at patient level and random slope for linear follow‐up time for the longitudinal component, (b) a Weibull model for the time‐to‐event component, (c) a current association specification of the association parameter α and an unstructured variance–covariance matrix. Patient‐specific outcome predictions according to *z*‐score trajectories were obtained using the Stata routine stjmcsurv [18] based on the dynamic prediction approach of Rizopoulos [19]. In a sensitivity analysis, investigating the association between a change in *z*‐score as a binary time‐dependent variable, we performed landmark analyses and time‐dependent Cox regression as previously described [20].

## 3 Results

### 3.1 Baseline characteristics of study population

Forty‐nine HR^+^/HER2^−^ breast cancer patients with a median age of 65.4 years [25th–75th percentile: 57.1–71.1] were included in this study. Main baseline characteristics are listed in Table 1. Most patients were female (*n* = 46, 94%) and had predominately invasive ductal tumors (*n* = 31, 63%), followed by invasive lobular tumors (*n* = 13, 27%). Our study cohort included 18 (37%) patients with *de novo* metastatic disease, two patients had locally advanced tumors (4%), and 29 patients (59%) had recurrent metastatic disease. Bone (*n* = 27, 55%) was the most frequent metastatic site, followed by lung (*n* = 17, 35%), then lymph nodes (*n* = 15, 31%), and liver (*n* = 8, 16%). One patient had brain metastases (2%). Twenty‐three patients (49%) had multiple metastatic sites at treatment start. The majority of patients (*n* = 46, 94%) received CDK4/6 treatment during the 1st or 2nd line of therapy. Patients were followed up for a median interval of 24.4 months [25^th^–75th percentile: 9.4–34.9]. During this follow‐up period, 24 (49%) patients progressed [median PFS: 15.7 months, 95% confidence interval (CI): 11.1–27.5], and 19 (39%) patients died (median OS: 27.8 months, 95% CI: 17.7‐not reached, Fig. S1).

**Table 1:** Baseline characteristics of total study population (*N* = 49). Distribution overall and by PFS event status. Data are medians [25th–75th percentile] for continuous data and absolute frequencies (%) for count data. *P*‐values were derived using Wilcoxon's rank‐sum, chi‐squared, or Fisher's exact tests. *P*‐values ≤ 0.05 are reported in bold.

	Total	No PFS event	PFS event	*P*‐value
	*N* = 49	*N* = 25	*N* = 24	
Age at inclusion (years)	65.4 (57.1–71.1)	65.9 (57.1–71.8)	64.5 (55.7–69.7)	0.873
Female gender	46 (93.9%)	23 (92.0%)	23 (95.8%)	1.000
Histological type				
IDC	31 (63.3%)	18 (72.0%)	13 (54.2%)	0.514
ILC	13 (26.5%)	5 (20.0%)	8 (33.3%)	
Other or not reported	5 (10.2%)	2 (8.0%)	3 (12.5%)	
Histological grade^a^				
Grade 1	4 (14.3%)	4 (30.8%)	0 (0.0%)	**0.022**
Grade 2	14 (50.0%)	7 (53.8%)	7 (46.7%)	
Grade 3	10 (35.7%)	2 (15.4%)	8 (53.3%)	
ECOG status at inclusion				
0	34 (69.4%)	20 (80.0%)	14 (58.3%)	0.084
1	14 (28.6%)	4 (16.0%)	10 (41.7%)	
2	1 (2.0%)	1 (4.0%)	0 (0.0%)	
Time from initial diagnosis to inclusion (months)	66.1 (1.2–133.3)	93.5 (1.0–168.2)	51.6 (14.8–108.5)	0.576
Time from initial diagnosis to metastatic disease (months)	68.1 (0.0–137.3)	98.4 (0.0–156.3)	64.3 (0.0–93.7)	0.316
Time from metastatic disease to inclusion (months)	1.0 (0.5–2.1)	0.9 (0.5–1.6)	1.2 (0.5–14.5)	0.523
*De novo* metastatic disease	18 (36.7%)	10 (40.0%)	8 (33.3%)	0.628
Number of metastatic sites^b^				
One	24 (51.1%)	13 (54.2%)	11 (47.8%)	0.664
Multiple	23 (48.9%)	11 (45.8%)	12 (52.2%)	
Bone	27 (55.1%)	13 (52.0%)	14 (58.3%)	0.656
Lung	17 (34.7%)	11 (44.0%)	6 (25.0%)	0.162
Lymph nodes	15 (30.6%)	10 (40.0%)	5 (20.8%)	0.146
Liver	8 (16.3%)	1 (4.0%)	7 (29.2%)	**0.023**
Pleura	8 (16.3%)	2 (8.0%)	6 (25.0%)	0.138
Other	5 (10.2%)	1 (4.0%)	4 (16.7%)	0.189
CDK4/6 treatment line				
1st line	39 (79.6%)	23 (92.0%)	16 (66.7%)	**0.056**
2nd line	7 (14.3%)	2 (8.0%)	5 (20.8%)	
3rd or 5th line	3 (6.1%)	0 (0.0%)	3 (12.5%)	
(Neo)‐adjuvant chemotherapy	19 (38.8%)	9 (36.0%)	10 (41.7%)	0.684
(Neo)‐adjuvant endocrine therapy	28 (57.1%)	12 (48.0%)	16 (66.7%)	0.187
Chemotherapy in the metastatic setting	4 (8.2%)	0 (0.0%)	4 (16.7%)	**0.050**
Endocrine therapy in the metastatic setting	9 (18.4%)	2 (8.0%)	7 (29.2%)	0.074
Continuous log *z*‐score at baseline	0.8 (0.4–1.8)	0.6 (0.3–1.2)	1.1 (0.6–2.0)	**0.038**
Binary *z*‐score at baseline				
*z*‐score < 3	30 (61.2%)	18 (72.0%)	12 (50.0%)	0.114
*z*‐score ≥ 3	19 (38.8%)	7 (28.0%)	12 (50.0%)	

^a^ Data on histological grade were missing in 20 patients.

^b^ Two patients with locally advanced disease were excluded.

Patients who developed progressive disease and/or died during follow‐up were more likely to have higher grade tumors, liver metastases, CDK4/6 treatment in 2nd or later lines, and chemotherapy prior to CDK4/6 treatment (Table 1). Higher baseline continuous mFAST‐SeqS log *z*‐scores (median log *z*‐score: 1.14 vs 0.60, rank‐sum *P* = 0.038), but not an elevated baseline *z*‐score, as defined by an established cutoff at ≥ 3, were associated with the occurrence of a PFS event. Other covariables were similarly distributed between patients who developed progressive disease/death or who remained event‐free during follow‐up.

### 3.2 Elevated baseline mFAST‐SeqS *z*‐score does not predict clinical outcome

At baseline before start of CDK4/6 treatment, the median mFAST‐SeqS *z*‐score was 2.26 [25^th^–75th percentile: 1.55–6.02]. There were 19 patients (38.8%) with mFAST‐SeqS *z*‐score levels ≥ 3, and these patients were more likely to have grade 3 tumors, liver metastasis, and chemotherapy prior to CDK4/6 treatment. All other variables were not associated with elevated mFAST‐SeqS *z*‐score levels (Table 2).

**Table 2:** Baseline characteristics by elevated mFAST‐SeqS *z*‐score. Data are medians [25th–75th percentile] for continuous data and absolute frequencies (%) for count data. *P*‐values were derived using Wilcoxon's rank‐sum, chi‐squared, or Fisher's exact tests. *P*‐values ≤ 0.05 are reported in bold.

	*z*‐score < 3	*z*‐score ≥ 3	*P*‐value
	*N* = 30	*N* = 19	
Age at inclusion (years)	66.8 (51.8–74.6)	65.3 (58.2–69.1)	0.984
Female gender	27 (90.0%)	19 (100.0%)	0.267
Histological type			
IDC	17 (56.7%)	14 (73.7%)	0.502
ILC	9 (30.0%)	4 (21.1%)	
Other or not reported	4 (13.3%)	1 (5.3%)	
Histological gradea			
Grade 1	3 (16.7%)	1 (10.0%)	**0.020**
Grade 2	12 (66.7%)	2 (20.0%)	
Grade 3	3 (16.7%)	7 (70.0%)	
ECOG status at inclusion			
0	23 (76.7%)	11 (57.9%)	0.246
1	7 (23.3%)	7 (36.8%)	
2	0 (0.0%)	1 (5.3%)	
*De novo* metastatic disease	11 (36.7%)	7 (36.8%)	0.990
Number of metastatic sites			
One	13 (46.4%)	11 (57.9%)	0.440
Multiple	15 (53.6%)	8 (42.1%)	
Bone	15 (50.0%)	12 (63.2%)	0.367
Lung	12 (40.0%)	5 (26.3%)	0.327
Lymph nodes	10 (33.3%)	5 (26.3%)	0.604
Liver	2 (6.7%)	6 (31.6%)	**0.043**
Pleura	6 (20.0%)	2 (10.5%)	0.458
Other	4 (13.3%)	1 (5.3%)	0.636
CDK4/6 treatment line	1.0 (1.0–1.0)	1.0 (1.0–2.0)	0.306
(Neo)‐adjuvant chemotherapy	12 (40.0%)	7 (36.8%)	0.825
(Neo)‐adjuvant endocrine therapy	16 (53.3%)	12 (63.2%)	0.498
Chemotherapy in the metastatic setting	0 (0.0%)	4 (21.1%)	**0.018**
Endocrine therapy in the metastatic setting	5 (16.7%)	4 (21.1%)	0.720

^a^ Data on histological grade were missing in 20 patients.

^b^ Two patients with locally advanced disease were excluded.

**Fig. 1:**
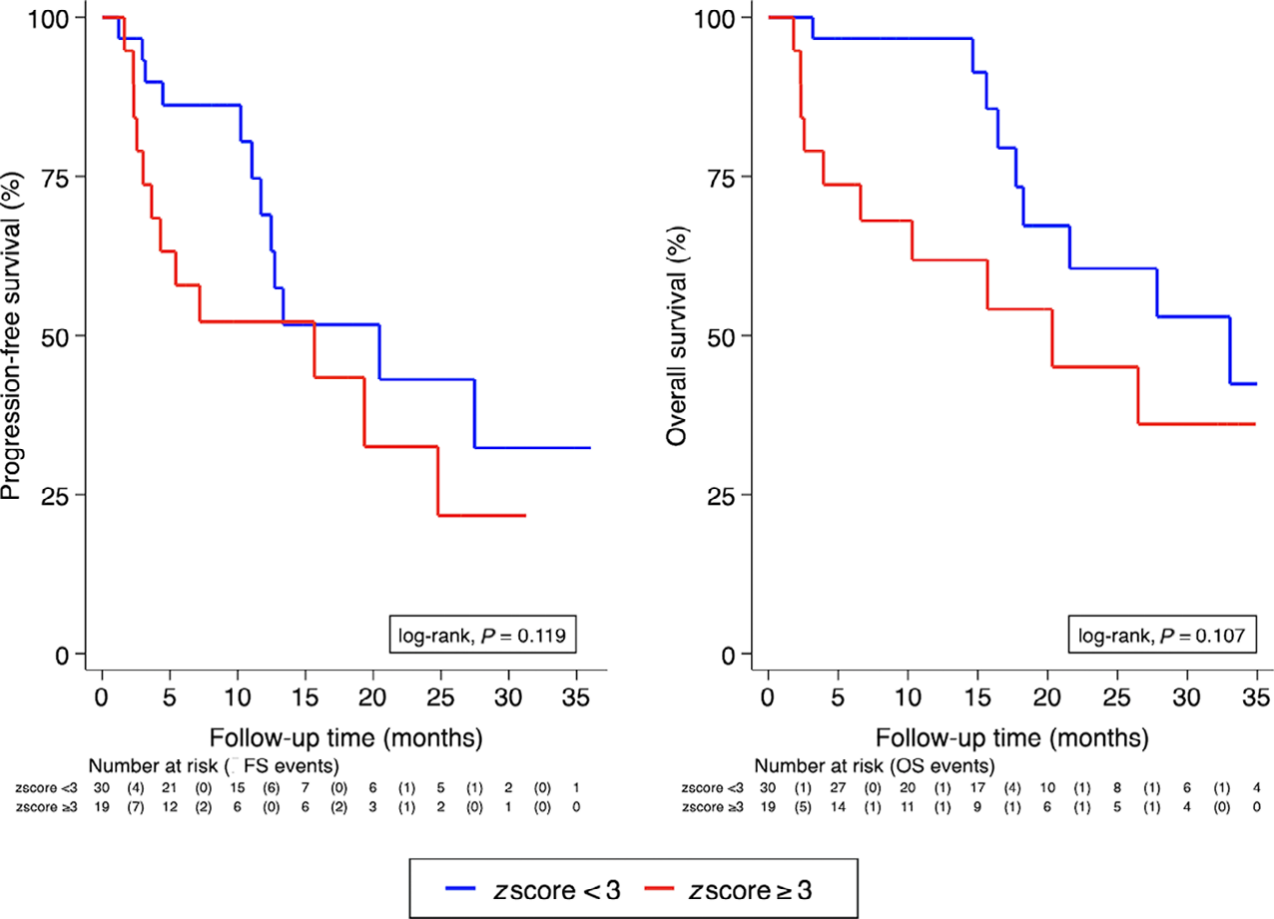
PFS (left panel) and OS (right panel) by elevated *z*‐score levels (cutoff ≥ 3) at baseline before start of CDK4/6 treatment (*N* = 49). Curves were estimated with Kaplan–Meier estimators. Significance was assessed by log‐rank test.

In univariable Cox regression analysis, neither baseline continuous log *z*‐scores nor baseline elevated *z*‐score as a binary variable were associated with PFS or OS (Fig. 1 and Table 3). Univariable predictors of worse PFS and/or OS included liver metastasis, multiple metastasis, CDK4/6 treatment in 2nd or later lines, and chemotherapy prior to CDK4/6 treatment (Table 3).

**Table 3:** Univariable baseline predictors of clinical outcome. HR, hazard ratio; Ref., reference group; NE, not estimable because no event occurred in male patients. *P*‐values ≤ 0.05 are reported in bold.

Variable	PFS				OS			
	HR	95% CI		*P*‐value	HR	95% CI		*P*‐value
Female gender	2.151	0.289	16.037	0.455	NE	NE	NE	NE
Histological type IDC	Ref.	Ref.	Ref.		Ref.	Ref.	Ref.	
Histological type ILD	1.404	0.579	3.405	0.453	1.031	0.377	2.820	0.952
Histological type Other	0.728	0.203	2.606	0.626	0.537	0.117	2.462	0.424
ECOG status (1 or 2 vs 0)	1.794	0.783	4.111	0.167	1.335	0.522	3.419	0.547
Histological grade (Grade 3 vs 1/2)^a^	2.486	0.899	6.877	0.079	2.767	0.873	8.771	0.084
*De novo* metastatic disease	0.948	0.401	2.236	0.902	0.945	0.357	2.501	0.909
Number of metastatic sites^b^	1.494	1.003	2.226	**0.048**	1.648	1.076	2.523	**0.022**
Bone	1.752	0.765	4.010	0.184	1.490	0.593	3.742	0.396
Lung	0.690	0.272	1.751	0.435	0.947	0.335	2.676	0.917
Lymph nodes	0.615	0.229	1.652	0.335	0.816	0.268	2.480	0.720
Liver	3.375	1.356	8.400	**0.009**	2.800	1.061	7.390	**0.038**
Pleura	1.834	0.713	4.716	0.208	2.171	0.812	5.808	0.123
CDK4/6 treatment line	1.869	1.254	2.786	**0.002**	1.800	1.100	2.944	**0.019**
(Neo)‐adjuvant chemotherapy	1.482	0.651	3.376	0.349	0.937	0.367	2.392	0.891
(Neo)‐adjuvant endocrine therapy	1.191	0.507	2.795	0.688	0.916	0.357	2.350	0.855
Chemotherapy in the metastatic setting	14.615	3.788	56.390	**< 0.001**	24.354	5.301	111.884	**< 0.001**
Endocrine therapy in the metastatic setting	2.403	0.988	5.845	**0.053**	1.114	0.367	3.382	0.848
Continuous *z*‐score at baseline	1.295	0.924	1.815	0.134	1.436	0.973	2.119	0.068

^a^ Data on histological grade were missing in 20 patients.

^b^ Two patients with locally advanced disease were excluded.

### 3.3 Longitudinal evolution of mFAST‐SeqS *z*‐score trajectories during CDK4/6 therapy in patients with and without PFS event

To investigate the longitudinal evolution of mFAST‐SeqS *z*‐scores under CDK4/6 therapy, we analyzed 181 *z*‐score measurements from 49 patients from baseline until the development of a PFS event or censoring alive without such an event (median number of measurements per patient = 3, 25^th^–75th percentile: 3–5, range: 1–8). Profiles of observed log *z*‐score measurements for patients with and without a PFS event are presented in Fig. S2.

**Fig. 2:**
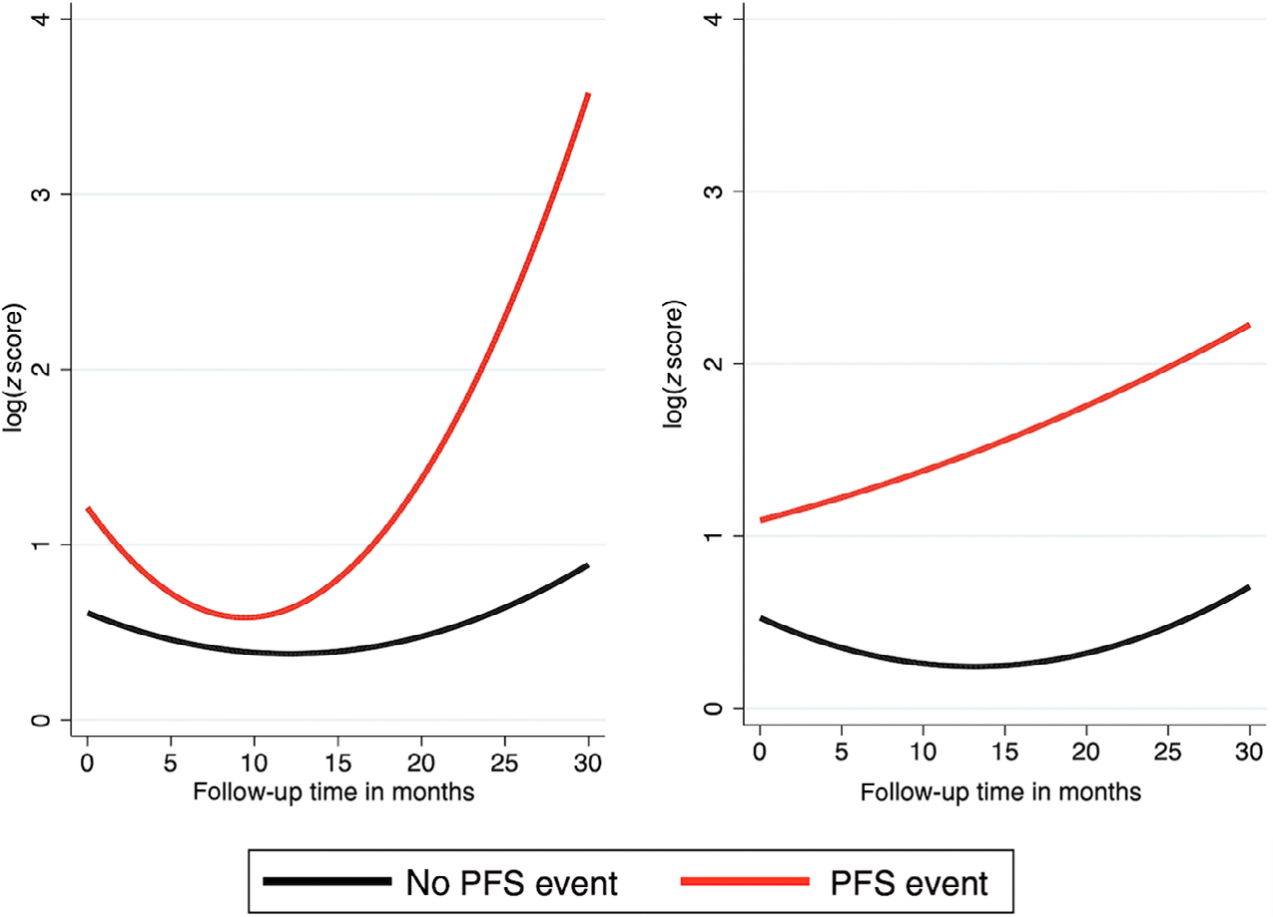
Predicted average longitudinal mFAST‐SeqS *z*‐score trajectories by PFS event status according to the mixed model (left panel) and the longitudinal component of the joint model (right panel). In the mixed model, missingness due to informative censoring results in over‐fitting of the model. In contrast, the joint model can simultaneously model the observed data and the missingness process, allowing for informative censoring to be corrected. Statistical analyses were performed by linear mixed regression (left panel) and joint model (right panel) and included 181 *z*‐score measurements form 49 patients.

First, we used a mixed model with a quadratic growth of mFAST‐SeqS *z*‐scores, a random intercept at the patient level, and a random slope for linear follow‐up time. According to this model, average baseline mFAST‐SeqS *z*‐scores were higher in patients who progressed than patients who did not progress (estimated difference 0.60, 95% CI: 0.132–1.068, *P*‐value = 0.012), and *z*‐score trajectories differed over time between patients with and without PFS event (*P*‐value for interaction with quadratic follow‐up time = 0.053, overall *P*‐value for difference in log *z*‐score over time = 0.110). Specifically, in patients without a PFS event, the average longitudinal mFAST‐SeqS *z*‐score levels remained relatively stable over time (Fig. 2). In contrast, in patients with a PFS event, average longitudinal mFAST‐SeqS *z*‐score levels decreased early on and then subsequently steeply increased. However, since this analysis does not account for potentially informative censoring and the missingness process, we next applied a joint model to account for this bias. In fact, when we used coefficients from the longitudinal component of the joint model, this pattern changed and became weaker, but differences remained statistically significant (Fig. 2). According to this joint model, patients who developed progressive disease had higher baseline *z*‐score levels (estimated difference 0.57, 95% CI: 0.147–0.983, *P*‐value = 0.008) and showed a slow but constant increase over follow‐up time, while patients without a PFS event had lower baseline *z*‐score levels and remained constantly low over follow‐up time (overall *P*‐value for difference in log *z*‐score over time = 0.024).

### 3.4 mFAST‐SeqS *z*‐score trajectories during CDK4/6 therapy are associated with clinical outcome

We then applied the joint model to study longitudinal *z*‐score trajectories and time to PFS event. In univariable joint modeling, patients with an elevated *z*‐score over time experienced a higher risk of a PFS event [HR of log *z*‐score at any time of follow‐up (i.e., the association parameter α = 3.3, 95% CI, 1.44–7.55, *P* = 0.005; Table 4)]. This finding is also consistent with Fig. 2. In multivariable analysis, adjusting the association parameter α for liver metastases, the association between an elevated *z*‐score over time and a higher PFS risk was observed similarly in patients with (adjusted HR per one unit increase in log *z*‐score = 3.6, *P* = 0.001) and without liver metastasis (adjusted HR per one unit increase in log *z*‐score = 2.9, *P* = 0.019, *P* for interaction = 0.576).

**Table 4:** Association of longitudinal log *z*‐score trajectories and time to PFS event using a univariable joint model with current value association structure. Wald‐test *P*‐value.

Trajectory variable	Association parameter α	95% CI	*P*‐value
log *z*‐score (per 1 unit increase)	3.3	1.44–7.55	0.005

In sensitivity analysis, neither time‐dependent Cox regression (time‐dependent HR = 2.1, 95% CI 0.82–5.21, *P* = 0.123) nor a landmark analysis (with landmark set at 6 months after initiation of CDK4/6 treatment, Fig. S3, Mantel–Byar test *P* = 0.117) indicated that a single change in *z*‐score level (from < 3 to ≥ 3) in a subsequent blood sample during follow‐up was a statistically significant predictor of an increased risk of developing a PFS event.

**Fig. 3:**
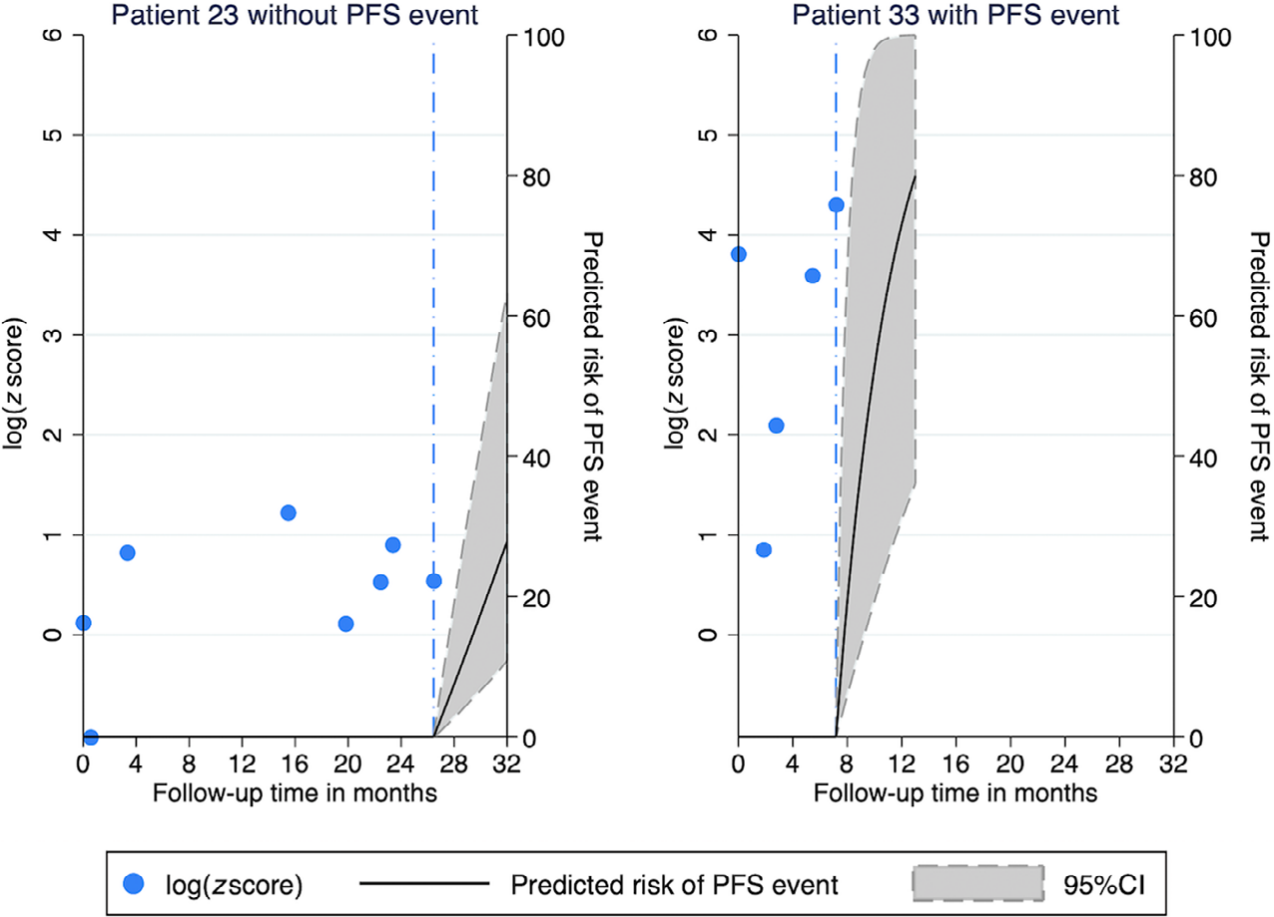
Personalized 6‐month risk predictions of progression or death for two patients according to their individual mFAST‐SeqS *z*‐score trajectory. Predictions were obtained from the joint model, which included the current value association structure. Blue dash‐dotted line: last study visit. PFS, PFS. Patient 23 had constantly low *z*‐score levels over follow‐up time, and his predicted risk of a PFS event 6 months after his last study visit was below 30%. Patient 33 had a high *z*‐score level at baseline, then a *z*‐score level decrease early on after initiation of CDK4/6 treatment, followed by a subsequent significant increase further on during treatment. His predicted 6‐month risk was above 75%.

Finally, personalized risk predictions of developing a PFS event conditional on individual patients' mFAST‐SeqS *z*‐score trajectories could be obtained with the joint model. Figure 3 illustrates this concept with two examples. Patient 23 had constantly low *z*‐score levels over follow‐up time, and his predicted risk of a PFS event 6 months after his last study visit was below 30%. Three months after his last *z*‐score measurement, the patient still showed no disease progression. Patient 33 had a high *z*‐score level at baseline, then a *z*‐score level decrease early on after initiation of CDK4/6 treatment, followed by a subsequent significant increase further on during treatment. His predicted 6‐month risk was above 75%. The patient developed progressive ascites 2 months after his last study visit.

## 4 Discussion

In this prospective study, we observed proof‐of‐concept that longitudinal ctDNA *z*‐score trajectories harbor important dynamic information on the development of disease progression in HR^+^HER2^−^ breast cancer patients undergoing CDK4/6 inhibitor treatment. By applying a so‐called joint model, we quantified changes of *z*‐score levels over time and estimated the relationship of these changes with the risk of developing progressive disease and/or death. We observed a constant increase of *z*‐score levels before development of disease progression, while *z*‐score trajectories remained constantly low over time in patients who remained event‐free. Moreover, the joint model revealed that elevated *z*‐score trajectories were significantly associated with higher progression risk. In contrast, single *z*‐score measurement at CDK4/6 inhibitor treatment initiation did not predict PFS risk. This shows that a longitudinal string of *z*‐score levels rather than a single measurement in time is required to obtain statistically significant predictions of outcome risk in this population.

Cancer is a dynamic disease, and the levels of a biomarker that reflect the underlying dynamic disease process are likely to change over time. By applying joint modeling, it is possible to use all longitudinally collected measurements during follow‐up time and to study how biomarker change through time during treatment, accounting for potential informative censoring and measurement errors [19]. Recent advances in joint modeling and the availability of adequate software packages helped to promote the application of this method in the field of cancer studies [10, 11, 12, 21, 22, 23, 24]. Our data demonstrate how this model can be applied to investigate the relationship between a longitudinal biomarker, that is, the *z*‐score as a surrogate for tumor fraction, and clinical outcome in cancer patients. As shown in Fig. 2, a mixed model of our data revealed higher baseline levels of *z*‐scores and a steep increase of biomarker levels over time in patients who progressed. However, applying a joint model to the same data and using the coefficients from the longitudinal component, the pattern of the trajectories changed. This is because the joint model accounts for informative censoring and thus reveals the true biomarker trajectories in patients with and without progressive disease. Finally, the joint model can also use the trajectories for providing dynamic time‐updated predictions for the individual patient. We could demonstrate this prediction by using *z*‐score trajectories of individual patients to estimate their future risk of disease progression (Fig. 3).

Modified Fast Aneuploidy Screening Test‐SeqS is a fast and cost‐effective untargeted approach, based on selective amplification of LINE1‐sequences across the genome, and allows detection of somatic copy number alterations (SCNAs) at a chromosome‐arm level [15]. In our previous study, we showed that quantitative changes of *z*‐scores within samples from the same patient showed a strong correlation with tumor fractions, suggesting that *z*‐scores present a robust measure for the longitudinal assessment of changing levels of ctDNA in individual patients. Several studies have shown similar results demonstrating that fluctuations of mutant allele fractions in plasma over time may show a relation to tumor response [6, 25, 26]. Our data also support the concept of ctDNA as a surrogate marker of disease burden, and that changes in ctDNA burden during treatment offer a potential for guiding clinical management of cancer patients. However, none of the published studies so far have analyzed the association of longitudinal biomarker measurements and time‐to‐event. In contrast to our previous study [8], we could not confirm the prognostic value of a single elevated baseline *z*‐score level in this patient cohort. Additionally, time‐dependent analyses did not suggest that a single change to an elevated *z*‐score level harbors enough prognostic information to predict PFS. Possible reasons for the discrepancy with our previous study include a more homogeneous patient cohort (HR^+^/HER2^−^, starting CDK treatment) and the fact that the majority of patients were at earlier stages of treatment in the present study compared to a more heterogeneous group of patients regarding biological subtype and number of treatment lines in the previous study. Although this inconsistency could be simply related to limited power due to our small sample size, this finding nonetheless illustrates how a longitudinal trajectory of biomarker measurements harbors meaningfully more prognostic information than a single measurement in time. Moreover, our joint model analysis implies that *z*‐score levels can be used at any time of follow‐up to predict risk of disease progression. This prognostic association also prevailed after multivariable adjustment for liver metastasis. Our data may suggest that CDK4/6 inhibitors can also be effective in patients with elevated *z*‐score levels as a surrogate for high tumor burden and that efficacy might be monitored frequently at low costs during treatment. This method might also prove helpful in future studies when addressing the question whether CDK4/6 inhibitors or chemotherapy are a better treatment choice in selected high‐risk patients.

Not all patients with progressive disease had elevated *z*‐score levels over follow‐up time, a fact that highlights a limitation of this technique. One reason is that the *z*‐score underestimates the tumor fraction in blood, when SCNAs are limited to few chromosomes [8]. Another reason might be that in some cases, there was a long time interval between the last *z*‐score measurement and the PFS event, and as such, an increase of log *z*‐score levels might have been missed. So ideally in future studies, the time interval should be kept as brief as possible to accurately investigate whether an increase truly contributes to a patient's risk. Another limitation of this study includes the small sample size, and therefore, this study needs validation in a larger data set.

## 5 Conclusion

In conclusion, this proof‐of‐concept study is the first to demonstrate that longitudinal assessment of tumor fractions using untargeted mFAST‐SeqS as a surrogate may harbor important prognostic information on disease progression in HR^+^/HER2^−^ breast cancer patients undergoing CDK4/6 treatment. Our data support the concept that longitudinally measured biomarkers such as ctDNA fractions rather than single time point measurements should be explored in future studies as a simple noninvasive biomarker for monitoring disease progression and treatment benefit in metastatic breast cancer patients.

## Conflict of interest

The authors declare no conflict of interest.

## Author contributions

ND and MB designed the study. RG performed sequencing experiments. CS, EVK, HDM, JL, AT, and MB contributed to data acquisition. ND and FP performed statistical analysis and interpretation of results. ND, MB, and FP drafted and revised the manuscript. ND, EH, and MB supervised the study. All authors reviewed the manuscript and approved its final version.

### Peer Review

The peer review history for this article is available at https://publons.com/publon/10.1002/1878‐0261.12870.

## Supporting information

**Fig. S1.** Progression‐free and OS experience of the total study cohort (*N* = 49). Curves were estimated with Kaplan‐Meier estimators.
**Fig. S2.** Line plot of log *z*‐score trajectories in patients (*N* = 49) who did not (left panel) and did (right panel) develop a PFS event during CDK4/6 treatment. Each dashed line represents the log *z*‐score trajectory of a single patient.
**Fig. S3.** Landmark analysis of predicted PFS according to patients (*N* = 49) who did or did not change to an elevated *z*‐score after 6 months of follow‐up (landmark time). Significance was tested by Mantel–Byar test.
**Table S1.** REMARK checklist.
